# Oral Surgical Management of Bone and Soft Tissues in MRONJ Treatment: A Decisional Tree

**DOI:** 10.3390/life10070099

**Published:** 2020-06-29

**Authors:** Antonia Marcianò, Erasmo Rubino, Matteo Peditto, Rodolfo Mauceri, Giacomo Oteri

**Affiliations:** 1 Department of Clinical and Experimental Medicine, University of Messina, 98124 Messina, Italy; antmarciano@unime.it; 2 Department of Biomedical and Dental Sciences and Morphofunctional Imaging, University of Messina, 98124 Messina, Italy; erasmo94@hotmail.it (E.R.); oterig@unime.it (G.O.); 3 Department of Surgical, Oncological and Oral Sciences, University of Palermo, 90127 Palermo, Italy; rodolfo.mauceri@unipa.it

**Keywords:** MRONJ surgical treatment, oral flaps, reconstructive surgery, MRONJ management

## Abstract

Background: The aim of the present work was to analyze a 10-year retrospective series of surgically treated medication-related osteonecrosis of the jaws (MRONJ) cases, reporting the clinical outcome and success rate for each adopted procedure in order to draw a treatment algorithm that is able to standardize clinical decision making and maximize the success of oral surgical treatment of MRONJ. Methods: Different surgical approaches were categorized taking into consideration two variables (a) hard tissue management (defined as debridement, saucerization or marginal resective surgery of maxillary necrotic bone) and (b) soft tissue management (defined as type of flap design and related modality of wound-healing). Results: For the retrospective cohort study, 103 MRONJ patients were enrolled and a total of 128 surgical procedures were performed. The role of radical-intended surgery using local flaps in MRONJ treatment was investigated, as well as palliative treatments. All stage I–II patients completely healed when a combination of radical necrotic bone surgery associated with a first intention healing of soft tissues was obtained. In stage III, when a patient was not eligible for maxillo-facial surgery, the use of palliative surgical strategies was effective in symptom relief in order to maintain a better quality of life for the duration of the patient’s life. Conclusions: Oral surgery with radical intent associated with a flap design able to ensure first intention healing might represent a valid option for the majority of MRONJ patients. The designed decision tree allows clinicians to assess individual surgical approaches for MRONJ treatment in accordance with patient-centered outcomes and surgical skills.

## 1. Introduction

Medication-related osteonecrosis of the jaws (MRONJ) is defined as exposed necrotic bone or bone that can be probed through a fistula in the maxillofacial region for at least 8 weeks in patients receiving an anti-resorptive medication for primary or metastatic bone cancer, osteoporosis, or Paget’s disease, without history of radiation therapy to the jaw [1]. Medication-related osteonecrosis of the jaw (MRONJ)’s official treatment guidelines are still lacking. Although the knowledge of diagnosis and staging of MRONJ has gradually advanced, and the most recent position papers provide detailed clinical and radiological diagnostic elements, a stepwise protocol for medical and/or surgical treatment has not been officially drawn [1,2].

To date, only a few stage-specific treatment indications deriving from the best practice of qualified centers for MRONJ prevention and treatment are available [3]. Nevertheless, these treatment protocols reported in the literature do not describe the step-by-step surgical procedures (from incision to suture techniques) to be used to favor wound-healing [4,5]. 

The present work aimed to critically analyze a 10-year retrospective series of surgically treated MRONJ cases, reporting the related clinical outcome and introducing a decision tree to identify the appropriate surgical approach. The purpose was either to (a) provide the surgeon with a treatment algorithm, based on MRONJ location and staging, that can aid and standardize clinical decision making; (b) increase the success rate of oral surgical treatments [6].

## 2. Materials and Methods

All study procedures were performed under the ethical standards of the Institutional and/or national research committee and the 1964 Helsinki declaration and its later amendments or comparable ethical standards. The study protocol was notified to the Ethical Committee of the Academic Hospital of Messina (Prot. n. 6311-24/03/2017) according to the current national law. This article does not contain any studies with animals performed by any of the authors [7]. 

In order to provide a clear description of the stage-related surgical procedures adopted in MRONJ treatment, a 10-year retrospective cohort study conducted at the Center for Treatment of the Osteonecrosis of the Jaws (University of Messina, Messina, Italy) was carried out.

Patient inclusion in the retrospective analysis required a diagnosis of MRONJ. 

MRONJ diagnosis was made according to the definition and staging system of the Italian Society of Maxillofacial Surgery (SICMF) and the Italian Society of Oral Pathology and Oral Medicine (SIPMO) [8,9]. Diagnosis was based on the following: (a) clinical findings of MRONJ (e.g., presence of exposed bone); (b) patients’ medical history reporting current or previous treatment with anti-resorptive or anti-angiogenic agents; (c) dental radiographies suggestive of MRONJ.

Patients were defined as stage I if they had focal MRONJ, that is, increased bone density limited to the alveolar bone region (trabecular thickening and/or focal osteosclerosis), with or without the following signs: markedly thickened and sclerotic lamina dura; persisting alveolar sockets; and/or cortical disruption in the presence of at least one minor clinical sign.

According to the SICMF-SIPMO definition, the minor clinical signs and symptoms are represented by the following: bone exposure; sudden dental mobility; nonhealing postextraction sockets; mucosal fistula; swelling; abscess formation; trismus; gross mandibular deformity and/or hypoesthesia/paranesthesia of the lips.

Patients were classified as stage II if affected by diffuse MRONJ when increased bone density was extended to the basal bone (diffuse osteosclerosis), with or without the following signs: prominence of the inferior alveolar nerve canal; periosteal reaction; sinusitis; sequestra formation; and/or oro-antral fistula observed at computed tomography (CT) imaging and the same clinical signs and symptoms as for Stage I.

Complicated forms were defined as Stage III if extra-oral fistula and/or displaced mandibular stumps and/or nasal leakage of fluids were present, and if computed tomography findings such as adjacent bone (zygoma, hard palate) involvement and/or pathologic mandibular fractures and/or osteolysis extending to the sinus floor were present.

Furthermore, the patients of all stages were classified into the subgroups of “a”—asymptomatic and “b”—symptomatic based on the presence/absence of pain and purulent discharge [8,9].

Characterization of the MRONJ cohort was made as follows: Patient demographics data were registered (age and gender); primary disease was analyzed, patients affected by metastatic malignant cancer disease (mainly carcinoma) were stratified by cancer type, i.e., breast cancer, lung cancer, prostate cancer, kidney cancer, etc. Patients affected by dysmetabolic disorders were divide into two categories of osteoporosis and rheumatoid arthritis. 

Suspected medications related to MRONJ onset and average duration of therapy in months have been reported.

MRONJ features such as anatomical location and stage frequency of the disease have been reported.

As previously reported [10], according to our center’s clinical routine, after MRONJ diagnosis the patients are included in a 8–10-week outpatient-based medical treatment program which includes local antiseptic administration (chlorhexidine and povidone iodide) and targeted antibiotic therapy. 

Regarding discontinuation of suspected medications, individual decisions should be made for every single case, even with respect to the drug-holiday protocol [11].

At the end of this initial phase the clinical course of MRONJ is evaluated and divided into four categories: progressive, unchanged, partially resolved and resolved. 

The clinical MRONJ course was categorized as reported by Watters et al. Patients were defined as progressive if they experienced an increase in swelling, discomfort, or secondary infection, or had radiographic evidence of worsening bone loss or destruction; unchanged if they continued to be symptomatic and had exposed necrotic bone with clinical or radiological results neither improving nor worsening; partially resolved if MRONJ lesions spontaneously improved or soft tissue coverage in the area was achieved with symptom improvement; and resolved when surgical wound-healing was achieved without dehiscence and without clinical signs of infection or evidence of recurrence. 

On the basis of the achieved outcome after medical treatment, the appropriateness of surgery with radical intent is discussed in a multidisciplinary meeting (MDM) where the chief oncologist and/or primary care physician are present.

For patients adamantly against receiving extensive surgery or those who do not want to undergo an invasive surgical procedure, the possibility of palliative surgical treatment is also discussed in the MDM.

Patients diagnosed with MRONJ (regardless of whether or not they are surgically treated [7]) are included in a follow-up routine program and recalled for clinical and radiological examination. The duration of follow-ups varies, generally being longer for patients affected by osteoporosis and shorter for cancer patients in consideration of a more limited life expectancy.

Only patients with at least 12 weeks of follow-up were included in the retrospective analysis.

To meet the objectives of this study, surgically treated patients were categorized according to the adopted surgical procedure taking into consideration two variables: (a) hard tissue management (defined as debridement, saucerization or marginal resection surgery) and (b) soft tissue management (flap design plus type of healing).

Surgical options entail conservative bone surgery (sequestrectomy and/or superficial debridement of sequestrum) and extensive bone surgery (alveoloplasty, resection). 

Patients treated with radical intent underwent radical removal of necrotic sequestrum determined through bleeding evidence of the surrounding bone (Figure 1, Figure 2, Figure 3, Figure 4, Figure 5, Figure 6, Figure 7, Figure 8, Figure 9, Figure 10, Figure 11, Figure 12, Figure 13 and Figure 14) [9]. In the absence of complete mucosal coverage of the surgically treated site, reintervention was performed [12].

Conservative surgical treatment, defined as palliative therapy, is usually adopted in stage III MRONJ forms when maxillo-facial surgery is contraindicated because of poor systemic conditions, and it consists of obtaining soft tissue relief from exposed bone irritation.

The clinical outcome was reported and defined as successful when surgical wound-healing was achieved without dehiscence and without clinical signs of infection or evidence of recurrence and functional recovery, as further prosthetic rehabilitation was viable to preserve the patient’s quality of life [4,5,13,14].

The decisional tree has been designed incorporating data of clinical outcomes of each single adopted procedure and following the high success rate registered. If success was obtained, the surgical procedure was suitable for that clinical scenario, and when healing was not achieved, the more appropriate surgical treatment option was defined through analysis of the re-entry procedure.

## 3. Results

For this retrospective cohort study, 103 MRONJ patients were enrolled and a total of 128 surgical procedures were performed. Patients’ clinical data were acquired, including sex, age and medical records to report any underlying primary diseases (cancer/osteoporosis).

Overall, the mean age was 70.72 years old. A female prevalence was observed (N = 76 subjects). A total of 49 patients received MRONJ-related medication for the treatment of dysmetabolic bone disease (N = 46 suffering of osteoporosis; N = 3 suffering of rheumatoid arthritis), and 54 were oncohematologic patients. 

In detail, of the 103 patients enrolled in the cohort, 11 patients had multiple myeloma, 24 had metastatic breast cancer, 16 had metastatic prostate cancer, 1 patient had metastatic lung cancer 1 had metastatic kidney cancer, and 1 had gastro intestinal stromal tumors (GISTs). 

Among the cancer patients, the most commonly administered medication was zoledronic acid (N = 37) followed by denosumab 120 mg (N = 13). Among this group of cancer patients, 1 subject was switched from zoledronate to denosumab 120 mg. Two patients consumed zoledronate plus pamidronate and one patient zoledronate plus ibandronate.

Among patients with dysmetabolic bone diseases, the most commonly administered bisphosphonate was alendronate (N = 28) followed by ibandronate (N = 9), risedronate (N = 2) and denosumab 60 mg (N = 2). One subject received clodronate. Two patients consumed alendronate and risedronate consecutively, and two were treated with alendronate followed by ibandronate. One patient with osteoporosis adopted a step-down therapy, switching from zoledronate to risedronate. One patient was treated with zoledronate followed by denosumab 60 mg. One patient consumed neridronate followed by ibandronate. 

As for cancer patients, the median onset of MRONJ was after 28.70 months in the case of zoledronate administration and after 26.92 months since starting a course of denosumab 120 mg. 

As for dysmetabolic patients, the average duration of antiresorptive therapy was 33.5 months in the case of denosumab 60 mg and 66.14 months in the case of alendronate, respectively. 

Characterization of the medication-related osteonecrosis of the jaw (MRONJ) cohort is reported in Table 1.

MRONJ features such as anatomical location and stage are reported in Table 2. The most common site of MRONJ was the mandible (N = 74). According to the SIPMO classification, the most frequent stage of MRONJ was stage II (stage IIa N = 45 lesions; stage IIb N = 11), whereas stage I (stage Ia N = 13 and stage Ib N = 27 lesions) and stage III (N = 15 lesions) were less common.

Among all the reported surgeries (N = 128), 113 were performed with radical intent (MRONJ stage I and II), while 15 procedures were palliative treatments (MRONJ stage III).

The different surgical approaches adopted with radical intent were analyzed and categorized as the following: superficial debridement (N = 16); sequestrectomy (N = 4); saucerization alias radical decortication of the necrotic bone (N = 22); sub-marginal bone resection (N = 25); marginal bone resection avoiding the loss of bony continuity (N = 46). 

Among this group, the most frequent surgery of choice was marginal bone resection.

As for soft tissue management, a mucoperiosteal flap was adopted in 29 surgical procedures; a coronally advanced mucoperiosteal flap in 57 surgeries. In advanced surgeries, to obtain tissue coverage, a mylohyoid flap (n = 23) or a pedicled buccal fat pad flap (n = 4) was needed.

The success rate was reported. A total of 113 procedures lead to a complete resolution of the disease. In 96 cases, healing was obtained with the first surgical treatment. Sixteen patients underwent a second intervention, and one a further third treatment to achieve complete and stable mucosal coverage.

Table 3 shows hard and soft tissue management details in the surgeries performed with radical intent in stage I and II MRONJ surgical procedures, detailing hard and soft tissue management with their related success rate.

Re-entry surgical procedures are reported in Table 4.

Palliative treatment was adopted in Stage III cases. A total 13 lesions were treated with superficial wound debridement, while in 2 cases a sequestrectomy (the removal of infected and avascular bone segments) was performed. Among the palliative treatments, partial healing was observed after eight procedures. Five lesions were unchanged and two worsened after treatment (Table 5).

A decisional tree was defined, in order to address the surgical options based on the extent, location, and stage of ONJ to provide an assessment tool useful to route the surgeon alongside such treatment options for standardization of the appropriate surgical procedures, clearly addressing both hard and soft of tissue management.

## 4. Discussion

The success rate of MRONJ surgical treatment proved to be high [2,15,16].

The surgical treatment raises questions regarding the margins of bone necrotic removal and the covering of the surgical wound by oral soft tissues, thus, a positive outcome is not always achieved [17,18,19,20].

The aim of the work was to demonstrate that adequate bone necrotic maxillary removal combined with different local flaps, usually adopted in oral reconstructive surgery, may prevent wound dehiscence, increase the vascularization, and protect the healing tissues against postsurgical opportunistic infections [21]. 

Primary flap closure is essential to promote oral mucosa healing in MRONJ patients, creating a more favorable environment for the effect of basal lamina signaling proteins [14]. These biochemical pathways resulted under-expressed in patients treated with drugs frequently involved in angiogenesis [22]. 

A previously reported cohort was expanded [6,12]. Moreover, oncohematologic cases were included, since all the treated patients received approval from the oncologist and underwent a safe surgery appropriate to their systemic condition. Both focal and diffused forms were included.

For patients with cancer, a decision regarding discontinuation of medications related to MRONJ should be based on the careful evaluation of benefits and toxicities in order to maximize their quality of life [23]. 

The scientific community seems to be divided. 

In a large follow-up study by Memorial Sloan Kettering Cancer Center (MSKCC), there was no statistical difference in the clinical course of MRONJ lesions in patients that discontinued intravenous bisphosphonates (BP) therapy when compared to those who had not suspended it [13].

Conversely, Hinson et al. reported that in case of BP administration independent of treatment modality and MRONJ stage at presentation, discontinuing BP before or at treatment initiation is associated with faster resolution of MRONJ symptoms compared to continuing the drug throughout jaw treatment. As assessed by the authors, patients should be counseled that continuing BP therapy after established MRONJ diagnosis may delay resolution of maxillofacial symptoms by approximately 4–6 months [24]. 

In a study by Ramaglia et al., results showed a significantly higher prevalence of completely healed sites in patients who followed the drug-holiday protocol [11].

In the experience of Hoefert et al. however, cessation of denosumab seemed to have no influence on healing outcomes in the non-surgical group and did not demonstrate any influence on surgical outcome [25].

However, all the patients of this cohort discontinued the suspected medication.

It is worth mentioning that in our country, discontinuation of denosumab is mandatory as expressly requested by the Italian Medicines Agency (AIFA) [26]. 

The role of radical-intended surgery using local flaps in MRONJ treatment was investigated as well as the palliative treatments. All stage I–II patients completely healed after radical surgery. Nevertheless, some cases required reintervention [27].

The analysis of the failed surgical procedures highlighted how both (a) the management of hard tissues to guarantee radical removal of necrotic bone and (b) the management of soft tissues to allow tension-free wound-healing influence the clinical outcome after surgery [28].

In every case of surgical failure, modifications to the management of soft tissue were required. Among these cases, additionally, three patients needed different hard tissue management (i.e., marginal resection instead of superficial debridement).

In stage III, when the patient was not eligible for aggressive maxillo-facial surgery, the use of palliative surgical strategies led to symptoms relief, allowing for the maintenance of maximum quality of life for the duration of their life.

Clinical experience from this case series provides important insight into MRONJ oral surgical treatment, highlighting that positive outcomes could be achieved with accurate presurgical planning.

The decisional tree, based on obtained results, allows clinicians to assess the degree of complexity required when selecting individual treatment approaches for MRONJ management, and to plan treatment in accordance with their surgical skills and the patients’ needs.

**Figure 15:**
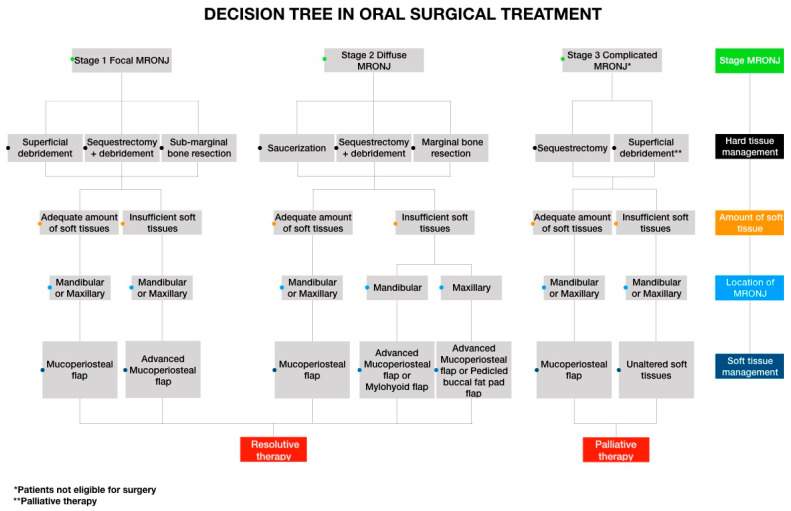
Decision tree.

Following the pathway of the decision tree (Figure 15), the surgeon must first locate the MRONJ lesion, given the different topographical anatomy of the maxillary and mandibular bone.

Subsequently, using the clinical/radiological stage, the surgeon will perform a risk/benefit assessment in terms of radicality of the intervention, deciding on a radical surgery with curative purposes in the focal and diffuse forms, and on palliative therapy in complicated forms or in patients with systemic conditions for which invasive surgery is contraindicated.

The surgical procedure most frequently used in the management of focal forms is the superficial debridement, whereas for diffuse forms, saucerization or a marginal bone resection is indicated.

In saucerization, the removal of the necrotic bone takes place using rotating drills and/or a piezoelectric device until obtaining the formation of a cup-like depression [2,29].

In marginal dental-alveolar bone resection, block removal of the pathological bone, avoiding the loss of mandibular bone continuity, is performed. This should always include an additional osteoplasty to eliminate residual bone asperity [30].

The choice between the two techniques is determined by the characteristics of the MRONJ lesion itself.

Palliative therapy through superficial debridement allows the removal of bone asperities giving relief to perilesional tissues for improved comfort of the patient; furthermore, the role of incomplete necrotic bone removal in terms of reduction in the local bacterial load in the case of suppuration refractory to antibiotic therapy has to be clarified.

The amount of perilesional soft tissue determines the choice of flap management [31].

The surgeon should perform a mucoperiosteal flap in cases with an adequate amount of soft tissues, or a coronally advanced mucoperiosteal flap in cases of insufficient amounts of perilesional soft tissues. In the advanced stages, a mylohyoid flap could be needed if the MRONJ lesion is localized in the jaw, while a flap pedicled buccal fat pad flap (PBFPF) may be needed if the lesion is located in the upper jaw [28,32].

In complicated forms, soft tissue management does not influence the clinical result, thus bone surgery with alveoloplasty and superficial debridement is performed to remove all bone asperities that generate ulcerative mucosal lesions in the patient. To promote a sense of well-being for the patient, the surgeon will opt for minimal soft tissue manipulation.

The use of platelet-rich plasma (PRP) rich in growth factors (PRGF) as well as the use of bone morpho-genetic proteins demonstrated an enhancement in vascularization and regeneration of osseous and epithelial tissues [33,34].

Nevertheless, given the high rate of successful healing achieved without any adjuvant intervention, we believe that the use of any bone stimulator, including all locally delivered growth factors such as PRP, PRGF and rhBMP, could be avoided.

Regarding the palliative treatment, this approach is adopted in Stage III cases to relieve symptoms and improve patients’ quality of life. Minimally invasive surgery techniques are chosen to limit patient involvement when major maxillo-facial surgical intervention is not viable for systemic contraindications.

Within palliation strategies, the removal of mobile sequestra is not considered a proper surgical act and is categorized within the medical conservative treatments. However, some MRONJ lesions without bone exposure may evolve into a sequestrum as results of compartmentalization by surrounding hard tissue. In these cases, the elevation of a flap is needed and, subsequently, the classification among surgical treatments.

## 5. Conclusions

The results from this retrospective study show that the surgical treatment of MRONJ is a reliable approach to the disease, and they confirm that appropriate soft tissue management procedures represent an additional guarantee against wound dehiscence, favoring the release of endogenous growth factors and leading to the successful healing of MRONJ disease.

Surgery with radical intent might represent a valid option for eligible MRONJ patients. The high clinical success rate achieved in the treated cases is due to an appropriate choice of surgical procedure, adjusted to case complexity.

Addressing the importance of promoting knowledge and, above all, the awareness that a comprehensive preoperative analysis of the hard and soft tissue components is essential to maximize the clinical result.

## Figures and Tables

**Figure 1:**
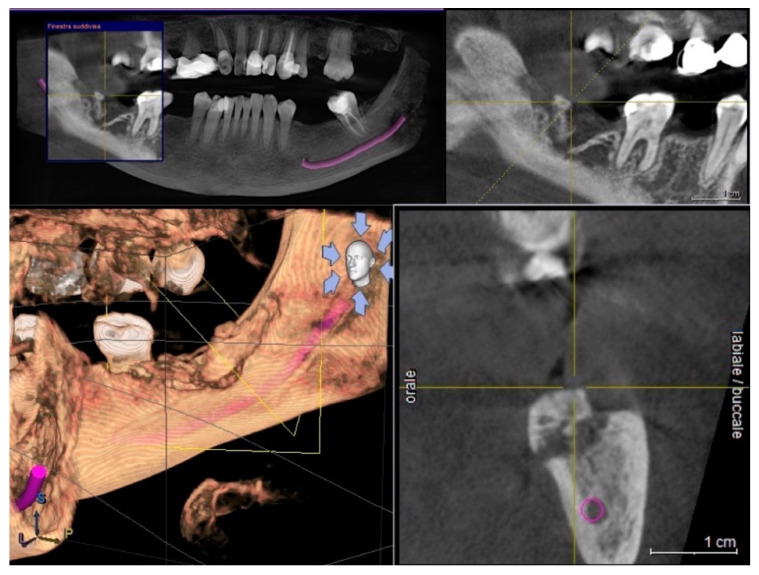
Cone beam-computed tomography showing the necrotic bone extension in a panoramic view with and without zoom, 3D rendering and cross-sectional view.

**Figure 2:**
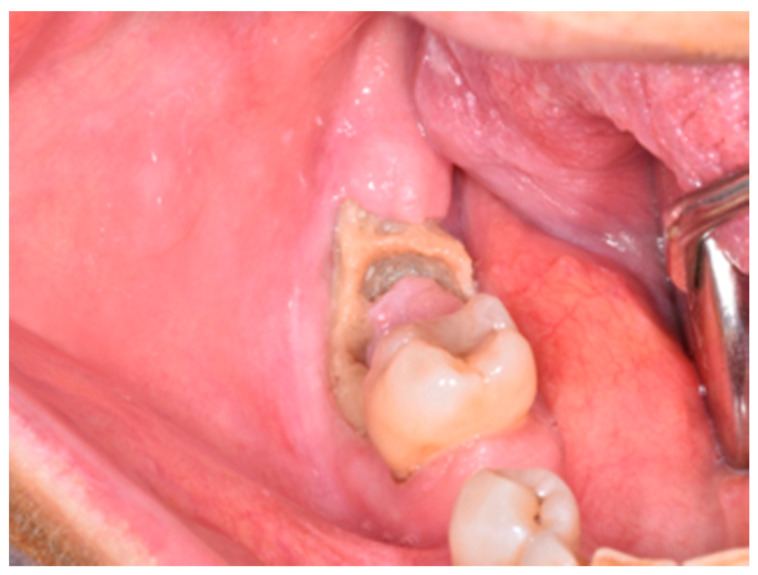
Clinical appearance of stage 1a MRONJ according to the the Italian Society of Maxillofacial Surgery (SICMF) and the Italian Society of Oral Pathology and Oral Medicine (SIPMO) staging system (absence of signs of infection/inflammation).

**Figure 3:**
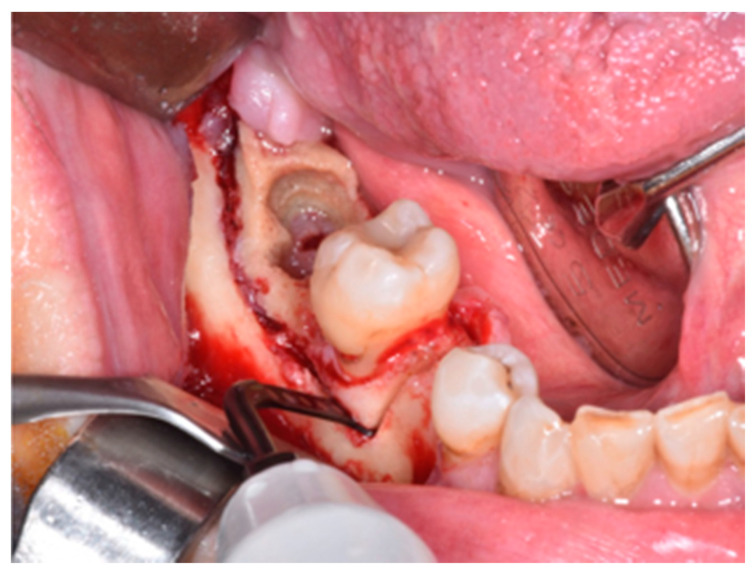
Sub-marginal resection performed using piezoelectric device.

**Figure 4:**
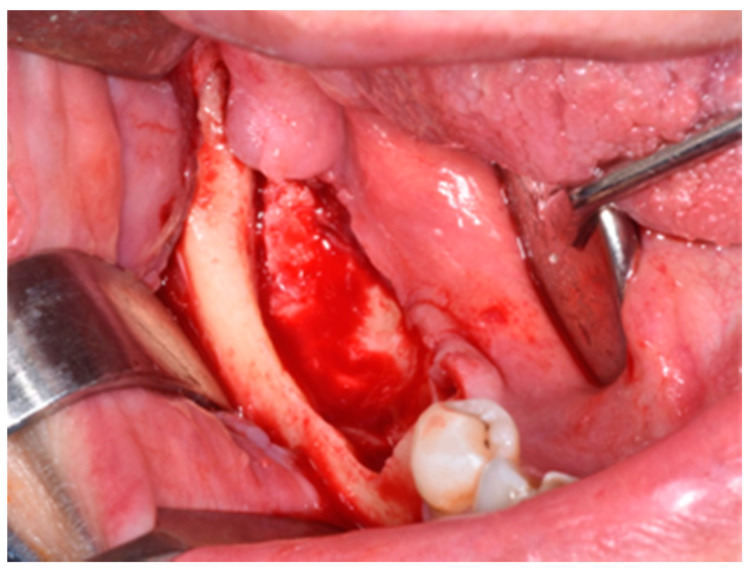
Revision of the cavity until observation of bleeding bone.

**Figure 5:**
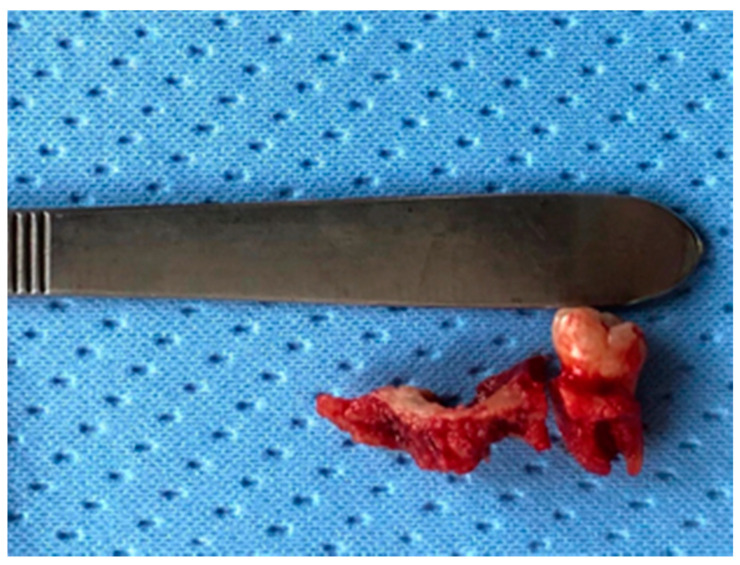
Resected sequestrum and extracted involved tooth.

**Figure 6:**
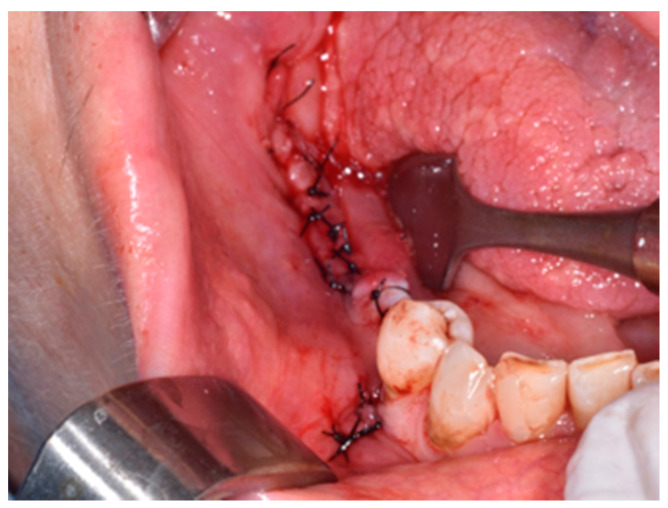
Suture with interrupted points to secure primary healing in order to avoid dehiscence.

**Figure 7:**
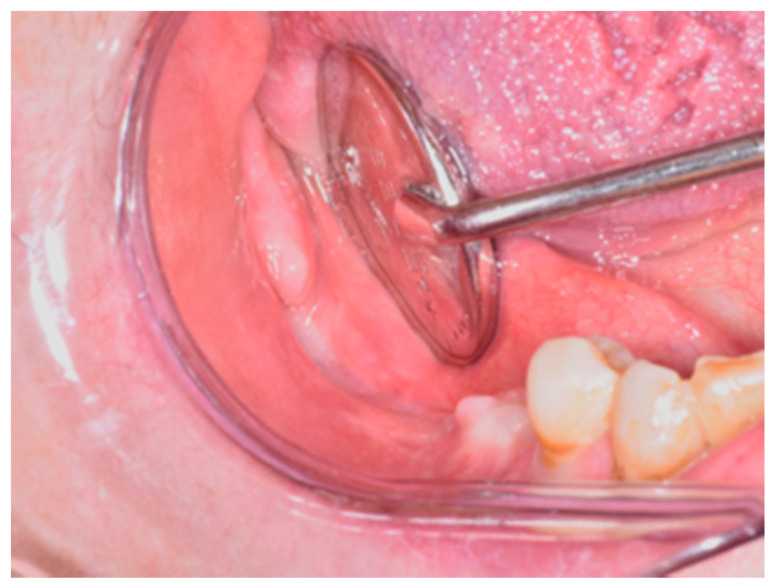
Healing without complications.

**Figure 8:**
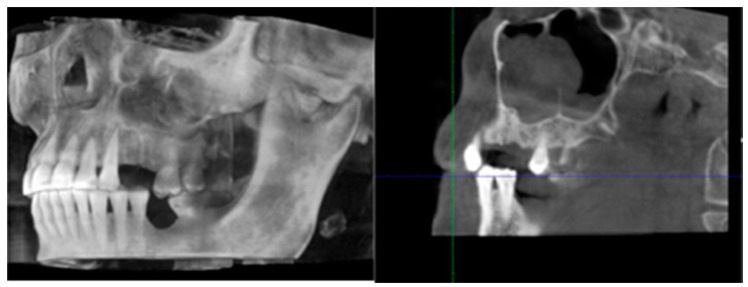
MRONJ “ghost socket” of the upper jaw as shown in 3D rendering, and coronal view of a cone beam computed tomography (CBCT) scan.

**Figure 9:**
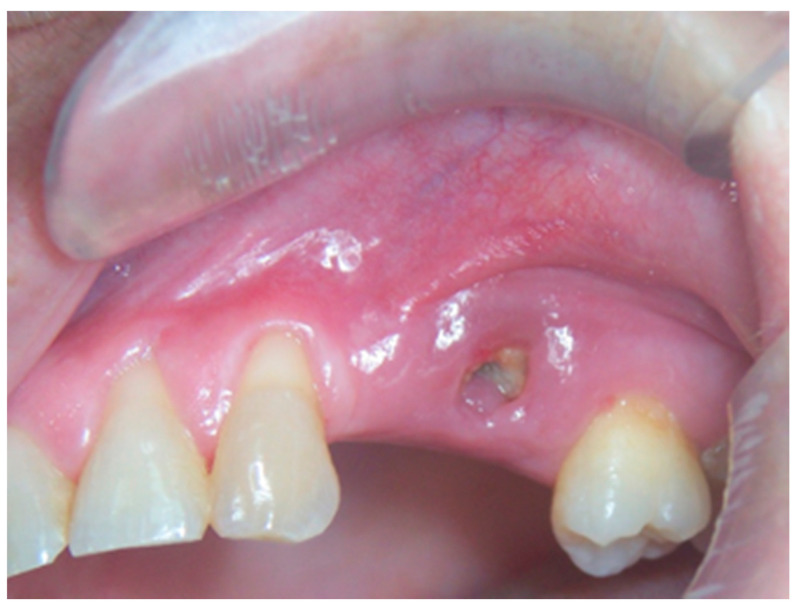
Maxillary osteonecrosis of the jaw at the time of diagnosis.

**Figure 10:**
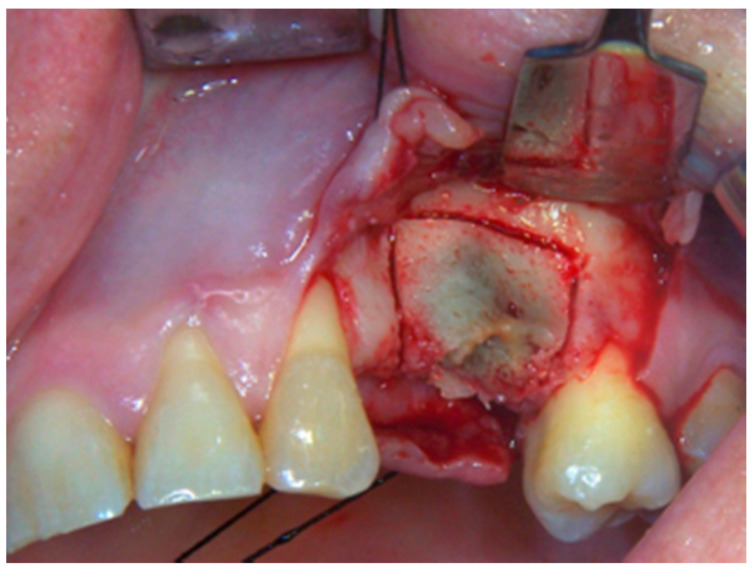
Intraoperative aspect.

**Figure 11:**
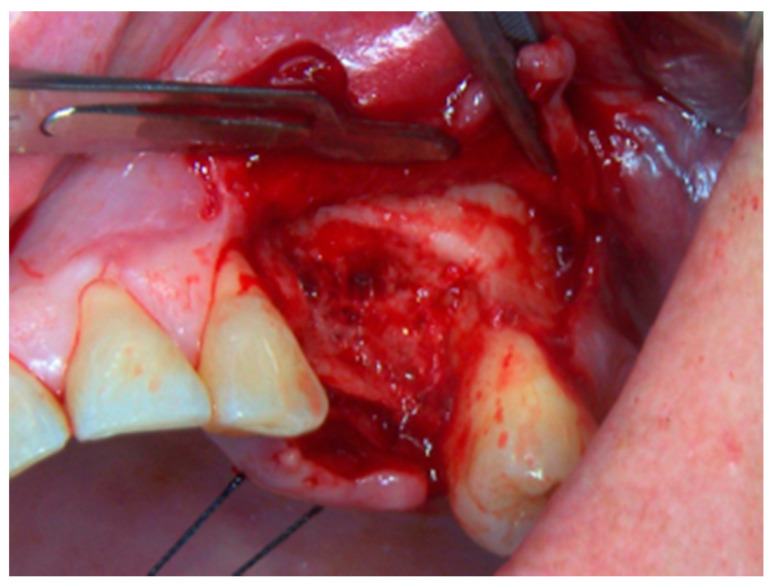
Intraoperative aspect after the removal of necrotic bone.

**Figure 12:**
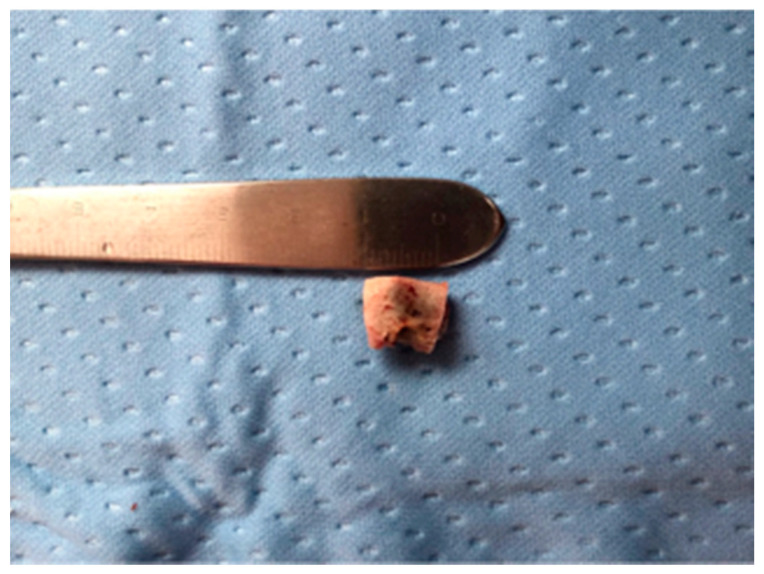
Resected bone segment.

**Figure 13:**
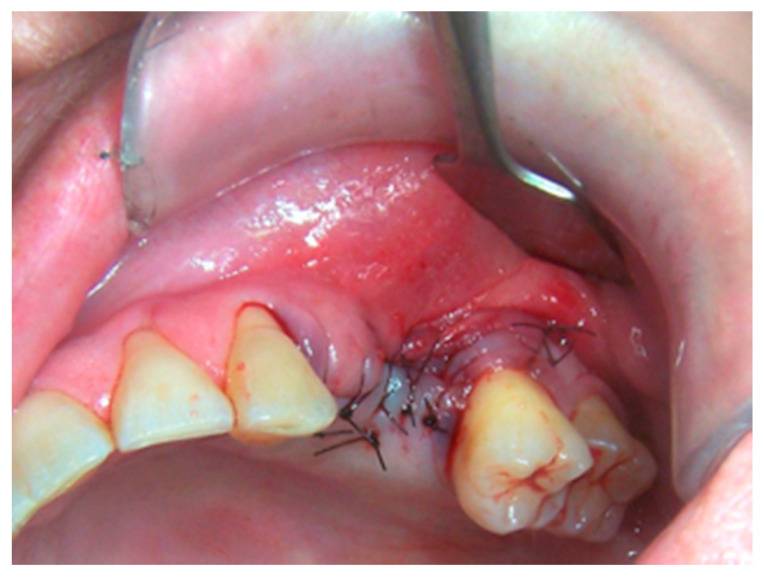
Tension-free advancement of the mucoperiosteal flap.

**Figure 14:**
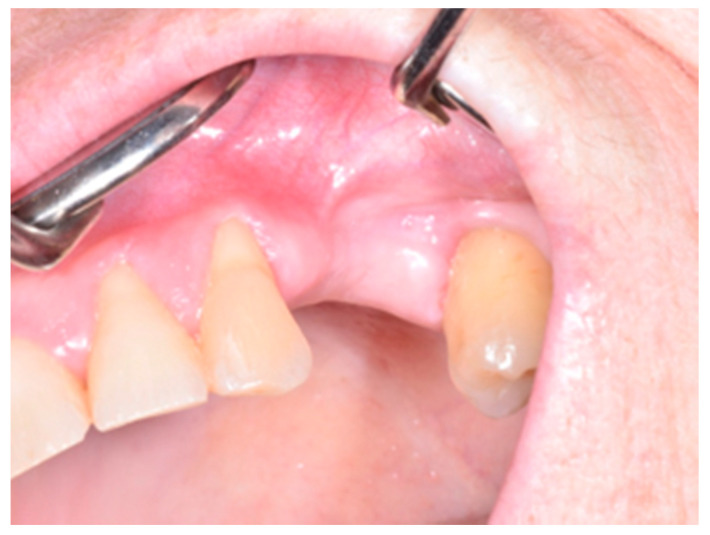
Evidence of mucosal healing.

**Table 1:** Characterization of the medication-related osteonecrosis of the jaw (MRONJ) cohort of 103 patients.

**Items**	**# (%)**
Age Years (Mean)	70.72
Sex	-
Male	27 (26.21%)
Female	76 (73.78%)
**Primary Disease**	**# (%)**
Breast cancer	24 (23.30%)
Prostate cancer	16 (15.53%)
Kidney cancer	1 (0.97%)
Multiple myeloma	11 (10.67%)
Lung cancer	1 (0.97%)
Gastrointestinal stromal tumors (GISTs)	1 (0.97%)
Rheumatoid arthritis	3 (2.91%)
Osteoporosis	46 (44.66%)
**Administered Antiresorptive Drug**	**# (%)**
Zoledronate (i.v)	37 (68.51%)
Denosumab 120 mg (subcutaneous)	13 (24.07%)
Denosumab 60 mg (subcutaneous)	2 (4.08%)
Alendronate (per os)	28 (57.14%)
Ibandronate (per os)	9 (18.36%)
Risedronate (per os)	2 (4.08%)
Clodronate (i.m)	1 (2.04%)
**Switch Therapy**	**# (%)**
Zoledronate (i.v) + Risedronate (per os)	1 (2.04%)
Zoledronate (i.v) + Pamidronate (i.v)	2 (3.70%)
Zoledronate (i.v) + Ibandronate (per os)	1 (1.85%)
Alendronate (per os) + Risedronate (per os)	2 (4.08%)
Alendronate (per os) + Ibandronate (per os)	2 (4.08%)
Neridronate (i.v) + Ibandronate (per os)	1 (2.04%)
Zoledronate (i.v) + Denosumab 120 mg (subcutaneous)	1 (1.85%)
Zoledronate (i.v) + Denosumab 60 mg (subcutaneous)	1 (2.04%)
**Average Duration of Therapy**	**Months**
Zoledronate (i.v)	28.70
Denosumab 120 mg (subcutaneous)	26.92
Denosumab 60 mg (subcutaneous)	33.5
Alendronate (per os)	66.14
Ibandronate (per os)	95.11
Risedronate (per os)	78.5
Clodronate (i.m)	24
Zoledronate (i.v) + Risedronate (per os)	113
Zoledronate (i.v) + Pamidronate (i.v)	52.5
Zoledronate (i.v) + Ibandronate (per os)	65
Alendronate (per os) + Risedronate (per os)	85.5
Alendronate (per os) + Ibandronate (per os)	164.5
Neridronate (i.v) + Ibandronate (per os)	70
Zoledronate (i.v) + Denosumab 120 mg (subcutaneous)	24
Zoledronate (i.v) + Denosumab 60 mg (subcutaneous)	99

Legend: # = number.

**Table 2:** MRONJ features (n = 103 subjects and n = 111 lesion).

Items	# (%)
Anatomic Location	-
Lower jaw	74 (71.84%)
Upper jaw	21 (20.38%)
Both jaws	8 (7.76%)
**SICMF-SIPMO Staging**	**Total (n = 111) (%)**
Stage 1a Stage 1b	13 (11.71%) 27 (24.32%)
Stage 2a Stage 2b	11 (9.90%) 45 (40.54%)
Stage 3	15 (13.51%)

Legend: # = number.

**Table 3:** Surgery with radical intent in stage I and II MRONJ.

Stage and Location of MRONJ	Bone Surgery					Flap Management				Surgeries # (%)	Success # (%)
	SD	SQ	SAU	SMBR	MBR	MPF	CAF	MYF	FPF		
Stage I Mandibular bone	✓	-	-	-	-	✓	-	-	-	5 (4.42%)	4 (80%)
	✓	-	-	-	-	-	✓	-	-	8 (7.07%)	8 (100%)
	-	-	-	✓	-	✓	-	-	-	7 (6.19%)	6 (85.71%)
	-	-	-	✓	-	-	✓	-	-	15 (3.27%)	13 (86.66%)
	✓	✓	-	-	-	✓	-	-	-	1 (0.88%)	1 (100%)
Stage II Mandibular bone	-	-	✓	-	-	✓	-	-	-	5 (4.42%)	2 (40%)
	-	-	✓	-	-	-	✓	-	-	7 (6.19%)	6 (85.71%)
	-	-	✓	-	-	-	-	✓	-	4 (3.53%)	4 (100%)
	-	-	-	-	✓	✓	-	-	-	5 (4.42%)	2 (60%)
	-	-	-	-	✓	-	✓	-	-	14 (12.38%)	11 (78.57%)
	-	-	-	-	✓	-	-	✓	-	19 (16.81%)	19 (100%)
	✓	✓	-	-	-	-	✓	-	-	1 (0.88%)	1 (100%)
Stage I Maxillary bone	✓	-	-	-	-	✓	-	-	-	1 (0.88%)	1 (100%)
	✓	-	-	-	-	-	✓	-	-	2 (1.76%)	2 (100%)
	-	-	-	✓	-	✓	-	-	-	1 (0.88%)	1 (100%)
	-	-	-	✓	-	-	✓	-	-	2 (1.76%)	2 (100%)
	✓	✓	-	-	-	✓	-	-	-	1 (0.88%)	1 (100%)
Stage II Maxillary bone	-	-	✓	-	-	✓	-	-	-	1 (0.88%)	0 (0%)
	-	-	✓	-	-	-	✓	-	-	3 (2.65%)	3 (100%)
	-	-	✓	-	-	-	-	-	✓	2 (1.76%)	2 (100%)
	-	-	-	-	✓	✓	-	-	-	2 (1.76%)	1 (50%)
	-	-	-	-	✓	-	✓	-	-	4 (3.53%)	3 (75%)
	-	-	-	-	✓	-	-	-	✓	2 (1.76%)	2 (100%)
	✓	✓	-	-	-	-	✓	-	-	1 (0.88%)	1 (100%)

Legend: # = number; SD = superficial debridement; SQ = sequestrectomy; SAU = saucerization; SMBR = sub-marginal bone resection; MBR = marginal bone resection; MPF = mucoperiosteal flap; CAF = coronally advanced mucoperiosteal flap; MYF = mylohyoid flap; FPF = pedicled buccal fat pad flap.

**Table 4:** Re-entry surgical procedures.

Patient #	1st Procedure						Re-Entry Procedure						
	Bone Surgery				Flap Management		Bone Surgery				Flap Management		
	SD	SAU	SMBR	MBR	MPF	CAF	SD	SAU	SMBR	MBR	CAF	MYF	FPF
1	-	✓	-	-	✓	-	-	-	-	✓	-	✓	-
2	-	✓	-	-	✓	-	-	-	-	✓	-	✓	-
3	-	✓	-	-	✓	-	-	-	-	✓	-	✓	-
4	-	-	-	✓	✓	-	-	-	-	✓	-	✓	-
5	-	-	-	✓	-	✓	-	-	-	✓	-	✓	-
6	-	✓	-	-	✓	-	-	✓	-	-	✓	-	-
7	-	-	-	✓	✓	-	-	-	-	✓	-	✓	-
8	✓	-	-	-	✓	-	✓	-	-	-	✓	-	-
9	✓	-	-	-	✓	-	-	-	✓	-	✓	-	-
10	-	-	-	✓	-	✓	-	-	-	✓	-	✓	-
11	-	-	-	✓	-	✓	-	-	-	✓	-	✓	-
12	-	-	-	✓	✓	-	-	-	-	✓	✓	-	-
13	-	-	-	✓	-	✓	-	-	-	✓	-	-	✓
14	-	-	✓	-	✓	-	-	-	✓	-	✓	-	-
15	-	-	-	✓	✓	-	-	-	-	✓	✓	-	-
16	-	-	✓	-	-	✓	-	-	-	✓	✓	-	-

Legend: # = number; SD = superficial debridement; SAU = saucerization; SMBR = sub-marginal bone resection; MBR = marginal bone resection; MPF = mucoperiosteal flap; CAF = coronally advanced mucoperiosteal flap; MYF = mylohyoid flap; FPF = pedicled buccal fat pad flap.

**Table 5:** Palliative surgical therapy of stage III MRONJ not eligible for maxillo-facial surgery.

Patient	Localization	Surgical Techniques			Outcome
		SD	SQ	MPF	
1	Maxillary	✓	-	✓	Partial Healing
	Mandibular	✓	-	-	Unchanged
2	Maxillary	✓	-	-	Unchanged
3	Mandibular	✓	-	✓	Worsened
4	Maxillary	-	✓	✓	Partial Healing
5	Maxillary	✓	-	-	Worsened
6	Mandibular	✓	-	✓	Partial Healing
7	Maxillary	✓	-	✓	Partial Healing
8	Maxillary	-	✓	✓	Partial Healing
9	Mandibular	✓	-	-	Unchanged
10	Mandibular	✓	-	✓	Partial Healing
11	Maxillary	✓	-	✓	Partial Healing
12	Maxillary	✓	-	-	Unchanged
13	Maxillary	✓	-	✓	Partial Healing
14	Maxillary	✓	-	-	Unchanged

Legend: # = number; SD = superficial debridement; SQ = sequestrectomy; MPF = mucoperiosteal flap.

## Abbreviations

MRONJ	Medication-related osteonecrosis of the jaws
BP	Bisphosphonates
SICMF	Italian Society of Maxillofacial Surgery
SIPMO	Italian Society of Oral Pathology and Oral Medicine
